# Height below 154 cm is a risk factor for pulmonary edema in twin pregnancy: An observational study

**DOI:** 10.1097/MD.0000000000040312

**Published:** 2024-11-08

**Authors:** Bingen Wan, Sheng Hu, Silin Wang, Yiping Wei, Jianjun Xu, Qiaoling Zheng

**Affiliations:** a Department of Thoracic Surgery, The Second Affiliated Hospital, Jiangxi Medical College, Nanchang University, Nanchang, Jiangxi Province, China; b Nanchang Medical College, Nanchang, Jiangxi Province, China.

**Keywords:** height, pregnant woman, pulmonary edema, twin pregnancy.

## Abstract

In recent years, twin pregnancies have become increasingly common. The aim of our study was to analyze the exposure to risk factors for postpartum pulmonary edema in twin pregnancies. We get all our data from the “DATADRYAD” database, which is available directly. We used a variety of statistical methods, including multivariate logistic regression analysis and smoothed curve fitting. The aim was to critically assess the relationship between height and the occurrence of postpartum pulmonary edema in pregnant women with twin pregnancies. Among pregnant women whose height was <154 cm, the risk of postpartum development of pulmonary edema gradually decreased with increasing height (OR = 0.65, *P* = .0104). There was no relationship between maternal height and postpartum development of pulmonary edema among pregnant women with height higher than 154 cm (*P* = .9142). Pregnant women who were taller than 154 cm had a 76% lower risk of developing pulmonary edema postpartum compared to pregnant women whose height was lower than 154 cm (*P* = .0005). Our study suggests that pregnant women with twin pregnancies whose height is <154 cm are more likely to suffer from postpartum pulmonary edema. Therefore, healthcare professionals and caregivers should pay closer attention to twin pregnancies with heights below 154 cm, be alert to the occurrence of pulmonary edema, and take preventive and therapeutic measures as early as possible. This will help prevent the development of pulmonary edema.

## 1. Introduction

Some studies have shown a continued increase in the rate of twin births, which is related to the improvement of medically assisted reproduction techniques and the postponement of the age of childbearing. However, these pregnancies not only increase the infant mortality rate, but also increase the risk of maternal complications (e.g., hypertension and pulmonary edema during pregnancy).^[1–4]^ And now the cause of combined pulmonary edema in twin pregnancies is not well understood, but maternal health is a global concern.^[5,6]^

Pulmonary edema is a common maternal complication and a significant contributor to maternal death.^[7]^ The pathologic change in pulmonary edema is the collection of excess fluid within the pulmonary interstitial and alveolar cavities,^[8]^ and its clinical manifestation is sudden dyspnea, which can be accompanied by irritability. Acute pulmonary edema can cause hypoxia in pregnant women, which can have serious consequences if not controlled in time.^[9]^

The use of ritodrine hydrochloride and corticosteroids, as well as hypertensive disorders of pregnancy, have been shown to have an impact on whether postnatal pulmonary edema occurs in pregnant women,^[10–12]^ but there is a gap in the current research on the relationship between maternal height and pulmonary edema in twin pregnancies. The aim of this study is to explore whether height is a risk factor for postpartum pulmonary edema in twin pregnant women, and to find relevant height thresholds to provide more diagnostic and treatment recommendations for twin pregnant women.

## 2. Materials and methods

### 2.1. Data sources

Data were obtained from the data-complete and authoritative “DATADRYAD” database (www.DATADRYAD.org). We have acquired data on pregnant women with twin pregnancies in this database through a routine process (https://doi.org/10.5061/dryad.1v8v6). The database file includes the following variables: age group, bed rest start, bed rest finish, period of bed rest, bed rest ≥ 6 weeks, pulmonary edema, height, weight, body mass index, obese, gestational age, term infant, gestational week, monochorionic, assisted reproductive technology, cesarean delivery, birth weight, pulmonary edema, blood loss, postpartum hemorrhage, blood transfusion, intraoperative transfusion, intraoperative transfusion ≥ 2000 mL, administration of magnesium, administration of corticosteroids, ritodrine start, ritodrine finish, period of administration, total dosage of ritodrine (mg).

### 2.2. Study population

This retrospective study collected obstetric data from 233 women with twin pregnancies who delivered between September 2009 and November 2016 at Central Hospital in Yamanashi Prefecture, Japan. Exclusion criteria were singleton death or twin death, severe fetal malformations, and women with double arterial perfusion sequences. The Dryad database is a public database and according to the regulations we can use its data directly without having to obtain informed consent from the patient again.

### 2.3. Statistical analysis

We analyzed the data using R software (version 3.6.3) and Empower Stats (version 4.2). Categorical variables were analyzed by chi-square test and continuous variables were analyzed by regression model. Statistical significance was considered when the *P* value was <.05.

To analyze the significance of the results between groups, the study used multiple regression equation analysis. To further analyze the correlation between height and postpartum occurrence of pulmonary edema in pregnant women with twin pregnancies, we used 3 models, Model Ⅰ: unadjusted for any variable; Model Ⅱ: adjusted for age, weight, bed rest 6 weeks or more; Model Ⅲ: adjusted for age, weight; bed rest 6 weeks or more, gestational week, term infant, monochorionic, assisted reproductive technology, cesarean delivery, sum of twin birth weights, pregnancy induced hypertension, postpartum hemorrhage, administration of magnesium, administration of corticosteroids, total dose of ritodrine (mg).

In addition, the correlation between height and postpartum occurrence of pulmonary edema in pregnant women with twin pregnancies was assessed using smoothed curve fitting and weighted generalized additive models.

## 3. Result

### 3.1. Twin pregnancies with height below 154 cm, the lower the height the more likely to get pulmonary edema

**Figure 1.**
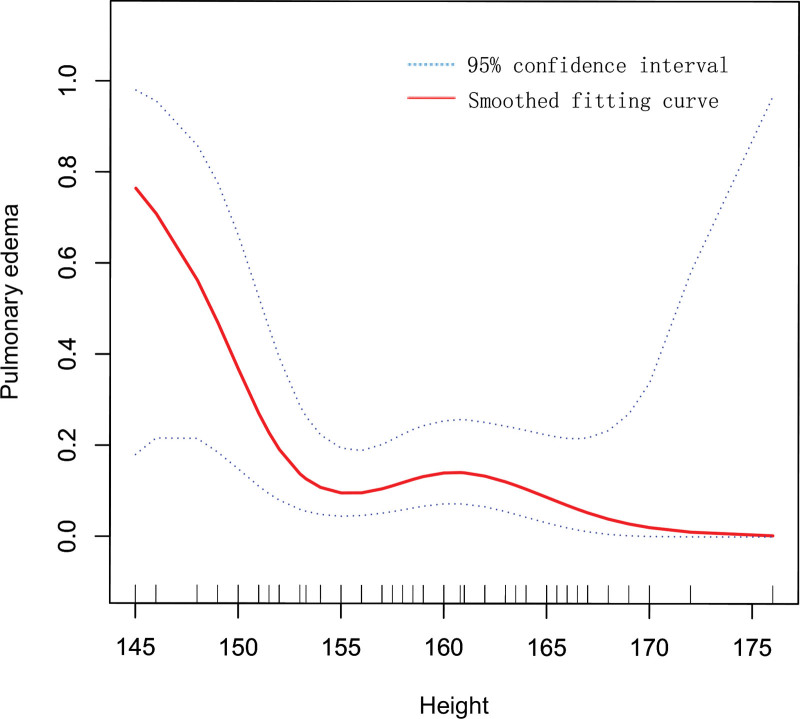
The relationship between height and pulmonary edema in pregnant women with twin pregnancies.

Since height is a continuous variable, it was necessary to perform threshold effect and saturation effect analyses (Table 1), and in this study we adjusted for age, weight, bed rest 6 weeks or more, gestational week, term infant, monochorionic, assisted reproductive technology, cesarean delivery, sum of twin birth weights, pregnancy induced hypertension, postpartum hemorrhage, administration of magnesium, administration of corticosteroids, total dose of ritodrine. The inflection point for height was found to be 154 cm, with a comparative likelihood ratio of 0.037. Before reaching the inflection point, the risk of postpartum development of pulmonary edema in pregnant women gradually decreased with increasing height (OR = 0.65, *P* = .0104), however, when crossing the inflection point, we observed that there was no relationship between the height of the pregnant women and postpartum development of pulmonary edema (*P* = .9142). To further understand the relationship between postpartum development of pulmonary edema and height in pregnant women with twin pregnancies, we performed smoothed curve fitting in this study (Fig. 1). We found the trend of the curves to be consistent with the results of the threshold effect and saturation effect analyses.

**Table 1:** Twin pregnancies with height below 154cm, the lower the height the more likely to get pulmonary edema.

Outcome	Pulmonary edema
	OR (95% CI) *P* value
Model I	
A straight-line effect	0.89 (0.80, 0.99) .0294
Model II	
Fold points (K)	154
<K-segment effect 1	0.65 (0.47, 0.90) .0104
>K-segment Effect 2	0.99 (0.86, 1.15) .9142
Effect size difference of 2 vs 1	1.52 (1.01, 2.30) .0461
Equation predicted values at break points	−2.00 (−2.77, −1.23)
Log likelihood ratio tests	0.037

Model II adjusted for the following variables: age, weight, bed rest 6 wk or more, gestational week, term infant, monochorionic, assisted reproductive technology, cesarean delivery, sum of twin birth weights, pregnancy induced hypertension, postpartum hemorrhage, administration of magnesium, administration of corticosteroids, total dose of ritodrine (mg).

CI = confidence interval, OR = odds ratio.

### 3.2. Higher incidence of maternal pulmonary edema in twin pregnancies below 154 cm in height

A sum of 226 patients with twin pregnancies were included in the study, 46 pregnant women with height <154 cm and 180 pregnant women with height >154 cm. The mean weight of pregnant women with height <154 cm was 46.79 kg and the mean weight of pregnant women with height more than 154 cm was 55.35 kg (*P* < .001). About 30.43% of twin pregnancies with height <154 cm developed acute pulmonary edema after delivery, while only 9.44% of twin pregnancies with height >154 cm developed acute pulmonary edema after delivery (*P* < .001). Intravenous supplementation with magnesium-containing fluids occurred in 18 of the pregnancies with heights <154 cm and in 29 of the pregnancies with heights >154 cm (*P* < .001). The difference between the total weight of twin fetuses was also statistically significant (*P* = .046). In other variables, no statistical differences were found. Baseline data for the study population are detailed in Table 2.

**Table 2:** Higher incidence of maternal pulmonary edema in twin pregnancies below 154 cm in height.

Groups	<154 cm (N = 46)	≥154 cm (N = 180)	Standardize diff. (95% CI)	*P* value
Height	150.78 ± 2.29	160.78 ± 4.39	2.86 (2.44, 3.28)	<.001
Weight	46.79 ± 6.32	55.35 ± 10.87	0.96 (0.63, 1.30)	<.001
Sum of twin birth weights	4057.26 ± 972.56	4388.28 ± 1003.04	0.34 (0.01, 0.66)	.046
Total dose of ritodrine (mg)	2079.97 ± 3985.88	954.40 ± 1923.23	0.36 (0.03, 0.69)	.081
Pulmonary edema			0.54 (0.22, 0.87)	<.001
No	32 (69.57%)	163 (90.56%)		
Yes	14 (30.43%)	17 (9.44%)		
Age			0.42 (0.10, 0.75)	.089
≤25 yr	8 (17.39%)	10 (5.56%)		
26–30 yr	13 (28.26%)	51 (28.33%)		
31–35 yr	12 (26.09%)	67 (37.22%)		
36–40 yr	12 (26.09%)	45 (25.00%)		
≥41 yr	1 (2.17%)	7 (3.89%)		
Bed rest 6 wk or more			0.24 (−0.09, 0.56)	.126
Yes	9 (19.57%)	20 (11.11%)		
No	37 (80.43%)	160 (88.89%)		
Gestational week			0.24 (−0.08, 0.57)	.147
<37 wk	28 (60.87%)	88 (48.89%)		
≥37 wk	18 (39.13%)	92 (51.11%)		
Term infant			0.23 (−0.09, 0.56)	.166
Yes	18 (39.13%)	91 (50.56%)		
No	28 (60.87%)	89 (49.44%)		
Monochorionic			0.07 (−0.25, 0.39)	.668
Yes	20 (43.48%)	72 (40.00%)		
No	26 (56.52%)	108 (60.00%)		
Assisted reproductive technology			0.34 (0.00, 0.68)	.07
Yes	4 (9.30%)	36 (21.43%)		
No	39 (90.70%)	132 (78.57%)		
Cesarean delivery			0.20 (−0.13, 0.54)	.256
Yes	40 (90.91%)	149 (84.18%)		
No	4 (9.09%)	28 (15.82%)		
Pregnancy induced hypertension			0.20 (−0.12, 0.53)	.196
Yes	9 (19.57%)	22 (12.22%)		
No	37 (80.43%)	158 (87.78%)		
Postpartum hemorrhage			0.19 (−0.13, 0.52)	.252
Yes	34 (73.91%)	117 (65.00%)		
No	12 (26.09%)	63 (35.00%)		
Administration of magnesium			0.53 (0.21, 0.86)	<.001
Yes	18 (39.13%)	29 (16.11%)		
No	28 (60.87%)	151 (83.89%)		
Administration of corticosteroids			0.29 (−0.03, 0.62)	.06
Yes	12 (26.09%)	26 (14.44%)		
No	34 (73.91%)	154 (85.56%)		

The results in the table are mean + SD/N (%). For continuous variables, use Kruskal–Wallis rank sum test. For count variables with theoretical number < 10, use Fisher’s exact probability test.

CI = confidence interval.

### 3.3. Pregnant women with twin pregnancies who are taller than 154 cm have a lower risk of developing pulmonary edema

A univariate analysis of the association between pregnant women with twin pregnancies and postpartum development of acute pulmonary edema showed that pregnant women with height >154 cm had a 76% lower risk of postpartum development of pulmonary edema relative to pregnant women with height <154 cm (*P* = .0005). Relative to pregnant women on bed rest for 6 weeks or longer postpartum, pregnant women on bed rest for <6 weeks had an OR of 0.28 (0.11–0.69) for developing pulmonary edema, a 72% reduction in the risk of postpartum pulmonary edema compared with pregnant women on bed rest for 6 weeks or longer (*P* = .0056). Relative to pregnant women with twin pregnancies with gestational hypertension, pregnant women without hypertension had an OR of 0.31 (0.13–0.76) for postpartum pulmonary edema, which was a 69% lower risk of postpartum pulmonary edema than pregnant women with gestational hypertension (*P* = .0104). Relative to pregnant women who developed postpartum hemorrhage, pregnant women who did not have postpartum hemorrhage had an OR of 0.26 (0.09–0.77), which was a 74% lower risk of developing pulmonary edema than pregnant women who had postpartum hemorrhage (*P* = .015). The corresponding OR, 95% CI and p-values for the study variables in this study are detailed in Table 3.

**Table 3:** Maternal risk of pulmonary edema is significantly lower in twin pregnancies than in twin pregnancies with heights below 154 cm.

Exposure	Statistics (%)	Pulmonary edema
		OR (95% CI) *P* value
Height	158.75 ± 5.71	0.87 (0.81, 0.94) .0003
Height		
<154	46 (20.35%)	Reference ^[1]^
≥154	180 (79.65%)	0.24 (0.11, 0.53) .0005
Age		
≤25 yr	18 (7.96%)	Reference ^[1]^
26–30 yr	64 (28.32%)	1.66 (0.33, 8.28) .5363
31–35 yr	79 (34.96%)	1.72 (0.36, 8.36) .4995
36–40 yr	57 (25.22%)	0.60 (0.10, 3.61) .5800
≥41 yr	8 (3.54%)	0.00 (0.00, Inf) .9912
Bed rest 6 wk or more		
Yes	29 (12.83%)	Reference ^[1]^
No	197 (87.17%)	0.28 (0.11, 0.69) .0056
Weight	53.60 ± 10.68	0.96 (0.92, 1.01) .1142
Gestational week		
<37 wk	116 (51.33%)	Reference ^[1]^
≥37 wk	110 (48.67%)	0.85 (0.40, 1.82) .6739
Term infant		
Yes	109 (48.23%)	Reference ^[1]^
No	117 (51.77%)	1.15 (0.54, 2.47) .7129
Monochorionic		
Yes	92 (40.71%)	Reference ^[1]^
No	134 (59.29%)	0.94 (0.44, 2.03) .8810
Assisted reproductive technology		
Yes	40 (18.96%)	Reference ^[1]^
No	171 (81.04%)	0.47 (0.19, 1.18) .1067
Cesarean delivery		
Yes	189 (85.52%)	Reference ^[1]^
No	32 (14.48%)	0.00 (0.00, Inf) .9884
Sum of twin birth weights	4320.91 ± 1003.70	1.00 (1.00, 1.00) .8806
Pregnancy induced hypertension		
Yes	31 (13.72%)	Reference ^[1]^
No	195 (86.28%)	0.31 (0.13, 0.76) .0104
Postpartum hemorrhage		
Yes	151 (66.81%)	Reference ^[1]^
No	75 (33.19%)	0.26 (0.09, 0.77) .0150
Administration of magnesium		
Yes	47 (20.80%)	Reference ^[1]^
No	179 (79.20%)	0.49 (0.21, 1.13) .0952
Administration of corticosteroids		
Yes	38 (16.81%)	Reference ^[1]^
No	188 (83.19%)	0.82 (0.31, 2.15) .6843
Total dose of ritodrine (mg)	1183.50 ± 2515.22	1.00 (1.00, 1.00) .0001

Data in the table: OR (95% CI) *P* value. Result variable: pulmonary edema; Exposed variable: height, age, bed rest for >6 wk, weight, gestational age, full-term infants, single chorion, assisted reproductive technology, cesarean section, the sum of twin birth weights, pregnancy induced hypertension, postpartum bleeding, administration of magnesium, administration of corticosteroids, total dose of ritodrine (mg); Adjust variable: none.

CI = confidence interval, OR = odds ratio.

### 3.4. Twin pregnancies with a height exceeding 154 cm have a lower risk of developing pulmonary edema

Multivariate regression modeling yielded a correlation between maternal height and postpartum pulmonary edema in twin pregnancies (Table 4), and found that pregnant women with a height >154 cm had a lower risk of postpartum pulmonary edema. Meanwhile, we built 3 models in Table 4. In Model Ⅰ, we did not adjust for any variables, and the OR for postpartum occurrence of pulmonary edema in pregnant women with height >154 cm was 0.24 (95% CI: 0.11–0.53, *P* = .0005), a 76% reduction in risk, relative to pregnant women with height <154 cm. In Model Ⅱ (adjusted for age, weight, bed rest 6 weeks or more), the OR for postpartum pulmonary edema was 0.22 (95% CI: 0.09–0.54, *P* = .0012) in pregnant women > 154 cm. In Model Ⅲ (adjusted for age, weight, bed rest 6 weeks or more, gestational week, term infant, monochorionic, assisted reproductive technology, cesarean delivery, sum of twin birth weights, pregnancy induced hypertension, postpartum hemorrhage, administration of magnesium, administration of corticosteroids, total dose of ritodrine), the OR for postpartum occurrence of pulmonary edema in pregnant women with height >154 cm was 0.21 (95% CI: 0.05–0.82, *P* = .0252).

**Table 4:** Twin pregnancies with a height exceeding 154 cm have a lower risk of developing pulmonary edema.

Exposure	Model I	Model II	Model III
	OR (95% CI) *P* value	OR (95% CI) *P* value	OR (95% CI) *P* value
Height	0.87 (0.81, 0.94) .0003	0.86 (0.79, 0.93) .0004	0.89 (0.80, 0.99) .0294
Height			
<154 cm	Reference ^[1]^	Reference ^[1]^	Reference ^[1]^
≥154 cm	0.24 (0.11, 0.53) .0005	0.22 (0.09, 0.54) .0012	0.21 (0.05, 0.82) .0252

Outcome variable: pulmonary edema. Exposure variable: height. Model I is no adjustment variable. Model II adjusted for the following variables: age, weight, bed rest 6 wk or more. Model III adjusted for the following variables: age, weight, bed rest 6 wk or more, gestational week, term infant, monochorionic, assisted reproductive technology, cesarean delivery, sum of twin birth weights, pregnancy induced hypertension, postpartum hemorrhage, administration of magnesium, administration of corticosteroids, total dose of ritodrine (mg).

CI = confidence interval, OR = odds ratio.

### 3.5. Stratified analysis of risk factors for pulmonary edema

Pregnant twins aged between 26 and 30 years had the risk of postpartum pulmonary edema (OR = 0.07, 95% CI: 0.02–0.32, *P* = .0006), with no statistical significance in other age groups. Pregnant women weighing between 55 and 110 kg (OR = 0.11, 95% CI: 0.01–0.82, *P* = .0313) and those undergoing a cesarean section (OR = 0.28, 95% CI: 0.12–0.66, *P* = .0035) are also at some risk of developing pulmonary edema after delivery. Twin pregnancies in which both fetuses have a total body weight of 4186 and 6078 g increase the risk of developing pulmonary edema, and the risk is higher in pregnancies with a total body weight between 4796 and 6078 g (OR = 0.21, 95% CI: 0.05–0.90, *P* = .0351). Higher risk of postpartum pulmonary edema when postpartum hemorrhage occurs (OR = 0.27, 95% CI: 0.11–0.66, *P* = .0038). Pregnant women on bed rest for <6 weeks (OR = 0.35, 95% CI: 0.13–0.90, *P* = .0302) have a higher risk of postpartum pulmonary edema compared to those on bed rest for more than or equal to 6 weeks postpartum (OR = 0.09, 95% CI: 0.01–0.56, *P* = .0102). Pregnant women with <37 weeks of gestation (OR = 0.28, 95% CI: 0.10–0.83, *P* = .0216) had a 9% higher risk of developing pulmonary edema postpartum than those with more than 37 weeks of gestation (OR = 0.19, 95% CI: 0.06–0.64, *P* = .0077). The risk of postpartum pulmonary edema is higher in part-term twin pregnancies (preterm or term pregnancies) than in full-term twin pregnancies (OR = 0.28, 95% CI: 0.1–0.82, *P* = .0203). The risk of postpartum pulmonary edema was higher in pregnant women who had unassisted births (OR = 0.24, 95% CI: 0.09–0.67, *P* = .006). Effect of the use of magnesium-containing drugs, corticosteroids and ritonavir on postnatal pulmonary edema in twin pregnancies. The OR, 95% CI and p-values for the other variables are detailed in Table 5.

**Table 5:** Stratified analysis of risk factors for pulmonary edema.

Subgroup	N	Pulmonary edema
X = height		OR (95% CI) *P* value
Age		
≤25 yr	18	0.00 (0.00, Inf) .9972
26–30 yr	64	0.07 (0.02, 0.32) .0006
31–35 yr	79	0.35 (0.09, 1.39) .1356
36–40 yr	57	0.79 (0.07, 8.31) .8412
≥41 yr	8	
Bed rest 6 wk or more		
Yes	29	0.09 (0.01, 0.56) .0102
No	197	0.35 (0.13, 0.90) .0302
Weight group		
38–47.5	63	0.42 (0.12, 1.43) .1633
48–54.7	85	0.28 (0.06, 1.30) .1035
55–110	78	0.11 (0.01, 0.82) .0313
Gestational week		
<37 wk	116	0.28 (0.10, 0.83) .0216
≥37 wk	110	0.19 (0.06, 0.64) .0077
Term infant		
Yes	109	0.19 (0.06, 0.65) .0081
No	117	0.28 (0.10, 0.82) .0203
Monochorionic		
Yes	92	0.25 (0.07, 0.86) .0282
No	134	0.23 (0.08, 0.66) .0064
Assisted reproductive technology		
Yes	40	0.05 (0.00, 0.62) .0195
No	171	0.24 (0.09, 0.67) .0060
Cesarean delivery		
Yes	189	0.28 (0.12, 0.66) .0035
No	32	1.00 (0.00, Inf) 1.0000
Sum of twin birth weights group		
1232–4164	75	0.81 (0.18, 3.57) .7790
4186–4792	75	0.05 (0.01, 0.25) .0002
4796–6078	76	0.21 (0.05, 0.90) .0351
Pregnancy induced hypertension		
Yes	31	0.37 (0.07, 1.91) .2346
No	195	0.22 (0.09, 0.56) .0016
Postpartum hemorrhage		
Yes	151	0.27 (0.11, 0.66) .0038
No	75	0.16 (0.02, 1.30) .0870
Administration of magnesium		
Yes	47	0.18 (0.04, 0.83) .0282
No	179	0.31 (0.11, 0.85) .0227
Administration of corticosteroids		
Yes	38	0.91 (0.14, 5.81) .9198
No	188	0.17 (0.07, 0.42) .0001
Total dose of ritodrine (mg) group		
384–15,916.8	150	0.14 (0.03, 0.56) .0057
NA	76	0.37 (0.13, 1.09) .0701

Data in the table: OR (95% CI) *P* value. Result variable: pulmonary edema; Exposed variable: height; Adjust variable: none.

CI = confidence interval, OR = odds ratio.

## 4. Discussion

Pulmonary edema in pregnancy and puerperium is a serious complication in pregnant women and an important clinical manifestation of uncompensated acute heart failure.^[9]^ The pathologic change in pulmonary edema is the collection of excess fluid within the pulmonary interstitial and alveolar cavities. Excessive fluid accumulation in the alveoli affects the function of alveolar surface-active substances and interferes with intra-alveolar gas exchange, which, if not corrected in time, will lead to hypoxemia, and in severe cases, even brain lesions.^[13–18]^ Despite the low incidence of maternal pulmonary edema, the mortality rate in patients with pulmonary edema is high, and pulmonary edema has a significant impact on maternal mortality in twin pregnancies.^[19,20]^ Pulmonary edema is mainly associated with increased hydrostatic pressure in pulmonary capillaries, plasma colloid osmotic pressure imbalance, increased endothelial cell permeability, lymphatic insufficiency or other factors.^[8,15,21–27]^

In this study, after adjusting for multiple variables and eliminating potential confounders, we found that postpartum pulmonary edema in twin pregnancies was associated with height, with a threshold height of 154 cm for twin pregnancies, and that twin pregnancies with a height below 154 cm had a higher probability of suffering postpartum pulmonary edema, and that the lower the height, the higher the probability of suffering postpartum pulmonary edema in twin pregnancies with a height below 154 cm The probability of postpartum pulmonary edema is higher in twin pregnancies below 154 cm.

After pregnancy, the fetus has an increasing need for nutrients and the mother’s body undergoes various physiological changes.^[28,29]^

To meet fetal growth, maternal cardiac output, blood volume and ventricular preload increase significantly,^[30–34]^ even ventricular muscle hypertrophy and compensatory cardiac remodeling.^[35–38]^ Previous studies have shown a higher increase in maternal cardiac output and blood volume in twin pregnancies compared to singleton pregnancies,^[36,37,39,40]^ which undoubtedly adds extra burden on the maternal cardiovascular system and reduces the compensatory capacity of the maternal heart. A study by Narang and Szymanski^[12]^ concluded that the higher the number of fetuses, the higher the incidence of gestational hypertension.

Maternal vascular resistance^[41,42]^ and hemoglobin concentration decrease during pregnancy,^[32,43]^ and studies have shown that the decrease in maternal vascular resistance is more pronounced in twin pregnancies.^[36]^ Factors such as increased blood volume and body water content will have a diluting effect on serum albumin and globulin,^[32,33]^ and increased excretion of albumin and urinary proteins,^[41]^ leading to a decrease in plasma colloid osmolality.

As the fetus grows, fetal compression of the pelvis and intra-abdominal organs becomes more pronounced. When the mother is in the supine position, there is significant compression of the inferior vena cava and a marked decrease in cardiac output.^[44–47]^ These changes lead to abnormal cardiac function, which impedes the return of blood to the lungs and ultimately leads to elevated pulmonary capillary pressures. These changes will make it easier for maternal intravascular fluid to escape into the interstitial and alveoli. Increased maternal chest circumference, elevated diaphragm, and decreased chest wall compliance during pregnancy,^[42,48]^ might decrease the intrathoracic volume and make the maternal lungs less likely to expand. The above physiologic changes will be more pronounced in mothers with twin pregnancies.

In combination with these factors, the probability of postnatal secondary pulmonary edema in pregnant women with twin pregnancies will increase.

There are not many studies on twin pregnancies, but the rate of twin pregnancies is increasing every year and our study fills part of the gap. Our study, which concluded that height is a risk element in postnatal pulmonary edema in twin pregnancies, is a wake-up call for physicians. For patients who are <154 cm tall and plan to have a twin pregnancy, physicians can more carefully assess whether the patient is suitable for a twin pregnancy. For the height of <154 cm, the twin pregnant women who are about to give birth to be more vigilant, prepare countermeasures in advance, be alert to the occurrence of pulmonary edema, and actively deal with the development of pulmonary edema.

There are limitations to our study. First, we used data from a public database and it was a cross-sectional study. Therefore, our study cannot clearly distinguish between causal relationships. Second, the results of the study would have been more convincing if there had been a larger number of subjects or more regions of participants. In addition, because it was a retrospective study, the effect of confounding factors could not be completely excluded, which would require further prospective studies to confirm this.

## 5. Conclusion

Our research shows that twin pregnancies with heights below 154 cm are more likely to develop pulmonary edema after delivery, and the lower the height, the greater the risk. Therefore, healthcare professionals and caregivers should pay closer attention to twin pregnancies with heights below 154 cm, be alert to the development of pulmonary edema, and take preventive measures and treatments as early as possible. This will help prevent the development of postpartum pulmonary edema.

## Acknowledgments

We are grateful that the DATADRYAD database is freely available to the public, and we honor those who collected the data.

## Author contributions

**Conceptualization:** Bingen Wan, Sheng Hu.

**Data curation:** Bingen Wan, Sheng Hu, Qiaoling Zheng.

**Formal analysis:** Sheng Hu, Silin Wang.

**Funding acquisition:** Sheng Hu.

**Investigation:** Jianjun Xu, Qiaoling Zheng.

**Methodology:** Bingen Wan, Sheng Hu, Silin Wang, Yiping Wei.

**Resources:** Sheng Hu, Silin Wang.

**Software:** Sheng Hu, Silin Wang.

**Supervision:** Yiping Wei, Qiaoling Zheng.

**Validation:** Bingen Wan, Silin Wang.

**Visualization:** Sheng Hu, Silin Wang.

**Writing – original draft:** Bingen Wan, Sheng Hu.

**Writing – review & editing:** Bingen Wan, Sheng Hu, Silin Wang, Yiping Wei, Jianjun Xu.
